# When caution becomes neglect: Deep brain stimulation and cognitive impairment in Parkinson's disease

**DOI:** 10.1177/1877718X261428282

**Published:** 2026-02-27

**Authors:** Simon JG Lewis

**Affiliations:** 1 Parkinson's Disease Research Clinic, Macquarie Medical School, Macquarie University, Sydney, Australia

**Keywords:** parkinson's disease, deep brain stimulation, cognitive impairment, patient selection, non-Motor symptoms, long-term outcomes

## Abstract

Over the past 25 years, a prevailing clinical dogma has held that significant cognitive impairment constitutes a contraindication to the use of Deep Brain Stimulation in Parkinson's disease. Whilst multiple studies, excluding such patients, have repeatedly emphasised the motoric benefits of this approach, less consideration has been given to the consequences of excluding this cohort. However, emerging evidence suggests that Deep Brain Stimulation in Parkinson's Disease patients with moderate cognitive impairment not only allows for significant reductions in dopaminergic therapy (typically alleviating many non-motor symptoms) but also favours survival and reduced admissions into institutional care. These small studies have not demonstrated significantly increased surgical or stimulation related complications in such patients and would suggest that further prospective studies, specifically evaluating this approach are warranted. Indeed, in the absence of a successful disease modifying therapy, exclusion from Deep Brain Stimulation often commits these advanced patients to a trajectory of ineffective pharmacological complexity. Alternative infusion therapies are associated with high discontinuation rates whilst escalating dopaminergic therapy, frequently exacerbates non-motor complications, including orthostatic hypotension, hallucinations, somnolence, and cognitive fluctuations. This flawed treatment strategy further accelerates functional decline, hospitalisation, and institutionalisation. Therefore, a reluctance or failure to offer Deep Brain Stimulation, where appropriate, may inadvertently consign patients to poorer long-term outcomes.

## Plain Language Summary

Should cognitive impairment be such an important exclusion from being offered Deep Brain Stimulation

Many Parkinson's Disease (PD) patients who would benefit from Deep Brain Stimulation are not being offered this therapy on the basis of their level of cognitive impairment. However, there is evidence to suggest that such an approach would be safe, improves long term survival and reduces the need for institutional care. This paper highlights the need to explore a change in our curren0t clinical practice.

## Introduction

### The dogma

By 2010 (for review see,^1^), the jury had returned, and the verdict was clear. Deep Brain Stimulation was not only safe but was effective in improving good ON time in patients with advanced Parkinson's Disease (PD). There were a few caveats that seemed reasonable, such as the need for a clear response to levodopa, exclusion of untreated or unresponsive psychiatric symptoms (e.g., psychosis, severe depression, impulsivity), exclusion of severe axial impairment (e.g., balance issues) and unfavourable neuroimaging (e.g., severe atrophy). However, at the same time, the presence of significant cognitive impairment was also promulgated into clinical practice as part of the DBS doctrine. Whilst the exclusion of those patients with dementia who lack capacity appears to have a clear rationale, the extension of this rule to a broader population of cognitively impaired patients, more likely reflected the constraints and eligibility requirements of the clinical trial protocols used in the initial landmark studies, rather than clear evidence of potential harm. Indeed, multiple studies have indicated that aside from a consistent drop on the assessment of verbal fluency following DBS, there is no evidence for an accelerated cognitive decline.^1^

### Sliding doors

A 69 year old patient attends his follow up appointment with his wife complaining of significant wearing off and dyskinesia. He has had 9 years of disease and is taking a monoamine oxidase inhibitor, a catechol-O-methyltransferase inhibitor, 200 mg of amantadine, the maximum dose of a dopamine agonist and 1000 mg of levodopa in a day. His wife raises concerns about a deterioration in his cognitive abilities over the past year and the patient reports some well formed but non-frightening visual hallucinations. His bedside testing reveals a Montreal Cognitive Assessment of 19/30.

### Door #1

Despite having no surgical contraindications for DBS, no referral was made to the local service due to concerns about his cognitive decline and future neuropsychiatric prognosis. The patient was assessed for all available infusion therapies. He did not want to consider intestinal gel preparations due to concerns relating to perceived high rates of device related complications, which can affect around 70% of cases.^2^ He developed more florid hallucinations with subcutaneous fos-levodopa, an adverse event that has been reported in 17% of patients within 12 months^3^ and was unable to tolerate subcutaneous apomorphine infusion due to skin nodules, which can affect over 50%.^4^ In an effort to improve motor symptoms, his levodopa dose was increased but this resulted in a fractured a hip when he fainted with orthostatic hypotension, reminding us all about the importance of screening for osteoporosis in this population.^5^ Hospitalisation to repair his fractured hip was associated with a prolonged delirium and a step decline in cognition. After extensive rehabilitation, the patient was discharged home but the reduced dose of dopaminergic therapy to control orthostatic hypotension, psychosis and somnolence resulted in poor mobility and caregiver stress associated with urinary incontinence. At the age of 70 years, the patient was transitioned into institutional care.

### Door #2

While it is impossible to know for any individual case whether a DBS pathway would have resulted in a better outcome, there is emerging evidence to suggest that we should avoid being overly cautious. In the first instance, it is well recognised that cognitive impairment represents a common part of disease progression in PD, which is not only associated with motor complications but commonly coexists with a range of non-motor symptoms. Furthermore, it is well recognised that many of these non-motor symptoms (e.g., psychosis, orthostatic hypotension, somnolence) can be exacerbated by increases in dopaminergic therapy and typically dose reduction forms a central part of their management.^6^ Therefore, it is possible that transitioning patients with motor complications to DBS could allow them to reduce their dopaminergic dose with a (partial/temporary) resolution of these coexistent non-motor symptoms, improving quality of life and reducing caregiver burden.

Many studies have previously reported that following DBS most patients can significantly reduce their levodopa equivalent daily dose (LEDD) by around 40% compared to baseline.^1^ Furthermore, a number of observational, long-term studies have reported that in patients with subthalamic nucleus DBS (STN-DBS), this LEDD lowering effect could be sustained for up to 10 years relative to their presurgical baseline^7^^.^^8^ However, what was less clear until recently was how the longitudinal trajectory of dopaminergic medication compared to those advanced patients with similar motoric impairment who did not undergo STN-DBS. Although, a retrospective study coming from a single site, Theyer et al. were able to compare 74 patients in an STN-DBS group to 61 medically managed patients who were matched for age of disease onset and age at the date of surgical baseline in the STN-DBS group.^9^ This control group was extracted from a random, prospective outpatient registry study, EuroPa^10^ and had undergone detailed serial assessments. Over 14 years, the STN-DBS group demonstrated a significant reduction in LEDD relative to their presurgical baseline (33.9–56.0%) and although stimulation levels increased over time, they did not predict dopaminergic dose reduction. In contrast, the LEDD in the non-surgical group rose by up to 50% compared to baseline with the greatest difference occurring at around 10 years before modest dopaminergic dose reductions were implemented in the more advanced stages of disease. The authors reported that STN-DBS was associated with a significantly lower number of patients taking dopamine agonists, although it should be appreciated that the EuroPa study was focused on Restless Legs Syndrome in PD, which might have influenced prescribing choices. Clearly, it could be argued that the medically managed group had ‘less aggressive’ disease and indeed, a faster escalation of LEDD in early PD has been reported as a predictor of future DBS.^11^ However, it is worth noting that both groups required similar levels of antipsychotic medication over the course of their disease progression, suggesting that they had comparable amounts of neuropathology. Despite the clear limitations of this small study, its findings would suggest that patients undergoing STN-DBS will require lower LEDD compared to the natural history of the disease. As noted above, many common non-motor symptoms associated with advanced PD are exacerbated by dopaminergic therapy and as such future studies should prospectively seek to identify if this LEDD reduction translates to lower non-motor symptom morbidity and improved quality of life for patients and their caregivers. It should also be highlighted that in patients where DBS targets the globus pallidus (GPi-DBS), reductions in LEDD are much less marked^1^ and this would need to be considered when designing such studies.

Concerns have been raised about the increased risk for surgical complications and prolonged hospitalisation following DBS in PD patients with significant cognitive impairment. However, several small studies have indicated that this risk may be overstated. Previously, some centres have considered GPi-DBS as a more appropriate approach for limiting the potential impact of post-surgical cognitive decline. One such study of GPi-DBS in 17 patients from a single centre, reported significant motor improvements (UPDRS III—41%, wearing off - 23%, dyskinesia - 69%) with no deterioration in neuropsychological performance over 6 months and transient postoperative confusion in just one patient.^12^ Another GPi-DBS study evaluating 25 patients over 3 years, reported similar motor improvements and stable cognitive performance at 12 months, which dropped at 3 years in keeping with the natural history of disease progression.^13^ The authors reported no immediate post-operative complications or prolonged hospitalisation. A more recent study has specifically sought to evaluate DBS in patients with moderate cognitive impairment including additional targets beyond the GPi (STN and the ventral intermediate nucleus of thalamus (VIM-DBS)).^14^ This study recruited two groups of patients undergoing DBS who were assessed on a comprehensive neuropsychological battery across domains of attention, executive function, memory, language, visuospatial performance and verbal fluency. Moderate cognitive impairment reflected performance that was two or more standard deviations below the normative mean on at least two test scores, whereas those patients with normal cognition had no more than one test in the battery that was more than one standard deviation below a normative score. In total, 40 patients with normal cognition and 40 with moderate cognitive impairment underwent DBS and were followed prospectively for 12 months. Significantly, following assessment it was determined that 25% of the cognitively impaired group actually satisfied diagnostic criteria for dementia (PDD). Notably, more patients with cognitive impairment underwent GPi or ViM targeting compared to STN-DBS, which was the most frequent target in the normal cognition group. However, significant motor improvements were the same between the two groups, as was the relative reduction in LEDD, despite the greater rates of GPi-DBS in the cognitively impaired group. Whilst the cognitively impaired cohort experienced a higher number of intracranial events and more instances of postoperative confusion and hallucinations, these differences did not reach statistical significance. Similarly, length of hospitalisation and the number of serious adverse events was equivalent between the two groups. However, transient cognitive deterioration was more frequent in the cognitively impaired group and one patient in this group experienced clinically significant cognitive decline following surgery, which was confirmed on repeat neuropsychological evaluation at 12 months.

Long-term survival and the transition to institutional care would represent key outcome measures that could help guide our clinical practice decisions. Some previous work has indicated that PDD patients undergoing DBS have higher rates of nursing home admission.^15^ However, this study did not include a control arm of matched patients who were conservatively managed, and the increased rates of institutionalisation were relative to DBS patients with normal cognition or mild cognitive impairment. More reassuring data comes from another single centre study that compared survival outcomes and nursing home admissions between 106 PD patients who had undergone STN-DBS with 41 patients who despite being good surgical candidates declined this opportunity.^16^ This allowed the groups to be well matched across age, gender, ethnicity, disease duration, LEDD and the key non-motor symptoms of depression. The study revealed that over a 10 year period, compared to those managed purely medically, the STN-DBS group had significantly longer survival (mortality rate 17% v 41%) and were significantly less likely to be admitted to a residential care home (admission rate 6% v 37%). It should be noted that the non-surgical patients did not undergo formal neuropsychological testing, which might represent a potential confound when interpreting this finding. However, the authors did include four patients who failed neuropsychometric assessment into an ‘intention to treat’ analysis, which did not impact the significant survival improvement in the DBS group. This study also reported that respiratory deaths were lower in DBS cases, which is significant given concerns about the long-term risk of bulbar impairment associated with this treatment. Indeed, previous work has suggested that STN DBS may reduce the risk of aspiration^17^ and potentially, the improved mobility in this group might also reduce the risk of death from respiratory complications.

## Future directions

Clearly, there is no evidence to suggest that DBS is disease modifying. However, it would appear that there is data to support DBS as a treatment that can improve survival and reduce the rates of institutional care. The immediate peri-operative risks associated with DBS in cognitively impaired PD patients does not seem to be greatly elevated. It is also likely that particularly STN-DBS can reduce the LEDD required to control motor symptoms over the long term with advancing disease. In turn, this might reduce common, comorbid non-motor features that lead to a reduced level of independence and poor quality of life. The argument could be made that a precision medicine approach should be adopted to further stratify risk in patient selection. For example, knowing if a patient has a more non-amnestic pattern of cognitive deficits (e.g., visuoperceptive) might allow for the more accurate prediction of the need for future residential placement.^15^ Similarly, given the known association between genetic risk and cognitive decline in PD (e.g., apolipoprotein E (APOE), β-glucocerebrosidase (GBA)),^18^ one might consider selected testing to allow a more nuanced conversation with our patients. However, in the absence of a treatment that could impact this trajectory towards dementia, perhaps we should focus on constructing more prospective studies that evaluate the benefits of DBS in PD with cognitive impairment, like the DBS-MODE that is currently ongoing.^19^ Ideally, such studies should be international and multi-centre, capturing the patient and caregiver perspective over the longer term. Similarly, there is a need to evaluate high-quality, real-world data as has been emphasised elsewhere.^20^ Such longitudinal observational data that could capture outcomes, safety, and patient selection in more heterogeneous, clinically representative populations than are often included in clinical trials would be invaluable. Parkinson's disease patients with significant cognitive deficits and those that are likely to progress rapidly to dementia will generally have a limited life expectancy and higher burdens of comorbidity.^21^ Thus, broadening DBS eligibility would need to be weighed against realistic expectations of long-term benefit, functional outcomes, and quality of life on an individual basis and we need more data to help guide these decisions. Clearly, the costs and access to DBS are not inconsequential problems, especially given the disparities that exist across differing health systems where issues of fairness, opportunity cost, and health-care system sustainability would need to be considered carefully. However, in many societies, the burden of DBS in advanced patients is clearly dwarfed by the demands of daily infusion therapy and even increased pill burden (dopaminergic and non-dopaminergic). Finally, it should be recognised that most PD patients are not managed in specialist centres and as such, messaging about the risks of DBS in cognitively impaired patients needs to be clarified. Whilst we must remain cautious, the time has come for the field to proactively explore the potential benefits of DBS in cognitively impaired PD patients and to avoid consigning them to poorer long-term outcomes.
